# Diagnosis of personality disorders in adolescents: a study among psychologists

**DOI:** 10.1186/1753-2000-7-3

**Published:** 2013-02-11

**Authors:** Elisabeth Martina Petronella Laurenssen, Joost Hutsebaut, Dine Jerta Feenstra, Jan Jurgen Van Busschbach, Patrick Luyten

**Affiliations:** 1 Viersprong Institute for Studies on Personality Disorders (VISPD), P.O. Box 7, 4660 AA, Halsteren, The Netherlands; 2 Department of Medical Psychology and Psychotherapy, Erasmus Medical Centre, Rotterdam, The Netherlands; 3 MBT Netherlands, Halsteren, The Netherlands; 4 Research Department of Clinical, Educational and Health Psychology, Department of Psychology, University of Leuven, Leuven, Belgium and University College London, London, United Kingdom

**Keywords:** Personality disorders, Adolescents, Psychologists, Online survey

## Abstract

**Background:**

Recent guidelines concerning the treatment of personality disorders (PDs) recommend diagnosing PDs in adolescents. However, it remains unclear whether these guidelines influence the current opinions and practices of mental health care professionals.

**Methods:**

Five hundred sixty-six psychologists completed an online survey concerning PDs in adolescents, of whom 367 professionals reported working with adolescents. The survey contained demographical questions (age, gender, profession, work setting) and specific questions related to PD in adolescence.

**Results:**

Although a majority of psychologists working with adolescents acknowledged the existence of PDs in adolescents (57.8%), only a small minority diagnoses PDs in adolescence (8.7%) and offers a treatment specifically aimed at targeting PD pathology (6.5%). Reasons for not diagnosing PDs in adolescence mainly concerned the belief that adolescent personality problems are transient (41.2%) and that the DSM-IV-TR does not allow diagnosing PDs in adolescence (25.9%).

**Conclusions:**

Although practice guidelines might have influenced clinicians’ opinions about PDs in adolescence, they have had little impact so far on routine clinical practice.

## Background

Mental health care professionals have traditionally been reluctant to diagnose personality disorders (PDs) in adolescents because of their supposed transient nature [1] and because of stigmatizing effects [2,3]. For example, Westen and colleagues [4] assessed how often clinicians diagnosed PDs in adolescents with personality pathology (N=296). Clinicians were first asked to provide their own categorical Axis II disorders of one patient. Second, clinicians received a checklist with all Axis II criteria in random order, and were asked to decide whether each criterion applied to the patient. The authors found that when clinicians were using their own categorical Axis II diagnoses, only 28.4% (N=84) of the patients was diagnosed with an Axis II disorder and almost all patients had only one PD. When using the checklist, 36.8% (N=109) of the patients was diagnosed with a cluster A PD, 54.4% (N=161) with a cluster B PD, and 41.2% (N=122) with a cluster C PD. Also, approximately 33% of the patients was diagnosed with more than one PD. A possible explanation for the difference is that clinicians at first hesitate to diagnose PDs in adolescents because they believe certain features of PDs are normative and not particularly symptomatic of a personality disturbance per se [4].

Another possible explanation is that Westen’s research took place before the publication of evidence-informed guidelines for diagnosing PD in adolescence. New research since then has indicated, for example, that borderline personality disorder (BPD) in adolescents is common and that the diagnosis of BPD can be measured with sufficient reliability and validity. Regarding stability, the diagnosis of BPD remained stable over time only for the most severe subgroup of adolescents; however it is possible that symptoms were reduced during the course of treatment [3]. This accumulated evidence has also informed recent guidelines [5], so the above findings may have influenced clinical practice.

More generally, PDs can be diagnosed reliably in adolescents [6], and are highly prevalent; prevalence rates range from 10 to 15% in this age group [7,8]. Furthermore, PDs in adolescents are extremely invalidating and may cause serious current and future distress in young people and their environment [1,9]. For this reason, Chanen and colleagues [10] proposed early detection and intervention of PDs in adolescence. In line with these developments, recent treatment guidelines support diagnosing PDs in adolescents starting at age 13 (e.g., NICE) [5]. However, it remains unclear to what extent scientific evidence and practice guidelines concerning PDs in adolescence have found their way into actual clinical practice. This study investigated psychologists’ opinions and practices regarding the diagnosis and treatment of PD in adolescents in the Netherlands and Belgium. Specifically, psychologists were asked whether they thought PDs existed in adolescents, and were also asked about their actual practices regarding the diagnosis and treatment of PDs in adolescence.

## Methods

### Participants

Participants were psychologists from the Netherlands and Belgium, recruited through their respective professional organizations (the Dutch Institute for Psychologists (NIP) and the Flemish Association for Clinical Psychologists (VVKP)). In April 2012 participants were sent an email which contained a link to a web-based survey. We aimed to gather 500 completed surveys. Participants received a small reward of 10 Euros when they completed the whole survey. This approach turned out to be a success: within three days more than 500 invitees had responded. In order to limit the cost of the rewards, and given that 500 responses were more than adequate to answer the research questions, the survey website was closed. At that time a total of 596 professionals out of approximately 3000 members had responded. Of these, 30 respondents (5%) did not complete all questions and were excluded, leaving 566 respondents. Four hundred twenty-nine respondents were female (75.8%), which is representative of the percentage of female mental health care professionals in the Netherlands [11]. The mean age of participants was 40.0 years (SD=11.7, range 22–67). One hundred fifty-five respondents worked in primary care (27.4%), 332 in secondary care (58.7%) and 79 in psychiatric hospitals (14.0%). The average number of years in clinical practice was 12.5 (SD=9.73, range 0–45). The majority of participants worked with adolescents (N=367; 64.8%), which was our main group of interest. The Dutch law does not require ethical permission for non-intrusive questionnaire-based research.

### Measures

The survey was introduced as a study on PDs in adolescents. The online survey consisted of demographical questions (age, gender, profession, work setting) and specific questions related to PDs in adolescence. Specifically, respondents were asked (a) whether they believe that adolescents can be diagnosed with a PD, (b) whether they actually diagnose PDs in adolescents, and if not (c) what their reasons are for not diagnosing PDs in adolescents, and (d) whether they offer a specialized treatment for adolescents with PDs. The response categories for not diagnosing a PD in adolescents were as follows: 1) adolescence is a stormy developmental phase and personality pathology in adolescence is transient, 2) diagnosing a personality disorder in adolescents is not allowed according to the DSM-IV-TR, 3) the diagnosis is stigmatizing, and 4) other; please specify.

## Results

The majority of psychologists (57.8%) agreed that PDs can be diagnosed in adolescents. Significantly more psychologists who work with adolescents believe that PDs can be diagnosed in adolescents (64%) compared to psychologists working with adults only (46.2%), (Chi-square=19.99, *p<* 0.001).

Yet, of psychologists working with adolescents, only 8.7% (32 participants) reported that they indeed diagnose PDs in adolescents if applicable, and only 6.5% (24 participants) offered a specialized treatment. Treatment methods most used for these adolescents were Mentalization-based Treatment (MBT, 25%), Emotion Regulation Training (ERT, 16.7%), Schema-focused Therapy (SFT, 12.5%), and Dialectical Behavior Therapy (DBT, 12.5%).

Reasons for not diagnosing PDs in adolescents that were most reported were: (a) adolescence is a stormy developmental phase and personality pathology in adolescence is transient (41.2%), (b) diagnosing a personality disorder in adolescents is not allowed according to the DSM-IV-TR (25.9%), (c) the diagnosis is stigmatizing (9%), and (d) a combination of the above reasons (6.6%).

Table 1 shows that significantly more male psychologists believe that PDs can be diagnosed in adolescents compared to female psychologists. However, regarding practice, there were no gender differences. Further, there were no age-related differences between respondents.

**Table 1:** Gender, age, opinion and practice concerning PDs in adolescents: Number of positive response/ total response

**Question**	**Male**	**Female**	**Chi Square**	**Age<40**	**Age≥40**	**Chi Square**
PDs can be diagnosed in adolescents	88/137	239/429	7.702	185/319	142/247	0.576
	64%	56%	P = 0.021	58%	57%	P = 0.750
I diagnose PD in adolescents myself	8/94	24/273	0.365	18/197	14/170	0.278
	9%	9%	P = 0.833	9%	8%	P = 0.870

Pertaining to the work setting, psychologists working with adolescents in psychiatric hospitals were the most likely to be convinced that PDs can be diagnosed in adolescents compared to psychologists working in primary and secondary care (Chi-square=14.91, *p<* 0.001) and were also most likely to diagnose PDs in adolescents themselves compared to psychologists working in primary and secondary care (Chi-square=39.50, *p<* 0.001).

## Discussion

This study showed that the majority (57.8%) of psychologists in the Netherlands and Belgium who participated in the study acknowledged the existence of PDs in adolescents. However, only a small minority of psychologists working with adolescents actually diagnoses PDs in adolescence (8.7%) and offers a specific treatment for PDs (6.5%). Psychologists working with the most severely disordered adolescents (i.e. those working in psychiatric hospitals) were most likely to diagnose a PD in these youngsters. Reasons for not diagnosing PDs in adolescents mainly concerned the belief that adolescent personality problems are transient and that the DSM-IV-TR does not allow diagnosing PDs in adolescence. As a result, personality pathology in adolescence might be underdiagnosed, which might in turn prevent referral to specialized treatments. For example, assuming that the presenting problems are transient developmental phenomena might lead to alternative, probably insufficiently helpful, treatment strategies that are often offered in low doses (e.g., social skill training to treat interpersonal problems) [12]. Similarly, conceptualizing personality problems in terms of Axis-I problems might lead to an accumulation of unsuccessful treatments targeting the supposed Axis-I problem.

The hesitation of clinicians to diagnose PDs in adolescents may be delaying the development of treatment models for this group. Currently, there is relatively little research on effective treatments for adolescents with PDs [13]. For example, as far as we know, there have not yet been any randomized controlled trials (RCTs) focusing solely on adolescents with a formal BPD diagnosis. However, there are studies on adolescents with BPD traits that give hopeful results and seem to confirm the need for specialized treatment in this target group. For example, Rossouw and Fonagy [14] recently presented the results of the first RCT investigating Mentalization-based Treatment (MBT) in self-harming adolescents. MBT was compared to treatment as usual, and appeared more effective in reducing self-harm and depression. In another RCT, Chanen and colleagues [10] compared the effectiveness of cognitive analytic therapy (CAT) with manualized good clinical care in adolescents with symptoms of BPD. They found a reduction of externalizing psychopathology in both groups, with some evidence that patients in the CAT group improved more rapidly. Both studies also suggest that treatments may be effective within a relatively short time span. More research on effective treatments for this group of patients is warranted because adolescents with PDs are at greater risk for having a broad range of problems than adolescents without PDs [4,8,9,15-17]. Furthermore, these adolescents have a greater risk of developing problems in adulthood [18-20].

Our findings showed that current guidelines [5] have had little influence on actual clinical practice. Although many psychologists and psychiatrists believe that PDs in adolescence exist, most of them do not diagnose PD in adolescents, nor do they offer specific treatments. More generally, the question may be raised whether the minimal impact of guidelines on clinical practice is related to the diagnosis of PD in adolescents only. It might reflect a broader problem concerning diagnosis and treatment of psychiatric disorders. A strength of this study is the large sample size. About one out of five registered psychologists completed the survey. We can therefore conclude that our data are probably representative of the opinions and practices of psychologists in Belgium and the Netherlands. Limitations of this study include generalizability to other countries, and the reliance on self-report rather than registrations of actual routine clinical practice.

## Conclusions

In summary, the reluctance of professionals to diagnose PDs in adolescents might hinder the development and dissemination of appropriate interventions for these youngsters.

## Competing interests

The authors declare that they have no competing interests.

## Authors’ contributions

EMPL was responsible for the coordination of the study and the writing of the manuscript. DJF participated in the design of the study, the statistical analyses, the interpretation of the data and the revision of the manuscript. JH participated in the design of the study, the interpretation of the data and the revision of the manuscript. JVB was involved in the design of the study and the revision of the manuscript. PL made substantial contributions to the interpretation of the data and the revision of the manuscript. All authors read and approved the final manuscript.
